# Rural and Indigenous Health Disparity in Medical Service Use for Dementia and Diabetes Mellitus in Minnesota

**DOI:** 10.1093/geroni/igaa057.829

**Published:** 2020-12-16

**Authors:** Sung Han Rhew, Patrick Bright, Andrine Lemieux, Wayne Warry, Kristen Jacklin

**Affiliations:** 1 University of Minnesota, Duluth, Duluth, Minnesota, United States; 2 Memory Keepers Medical Discovery Team, University of Minnesota, Duluth, Duluth, Minnesota, United States; 3 Memory Keepers Medical Discovery Team, University of Minnesota, Duluth, Minnesota, United States

## Abstract

Minnesota has shown relatively high growth of mortality from diabetes mellitus (DM) and dementia in recent years, especially in rural areas. Analysis of medical care utilization patterns may reveal the reasons for this trend. The goal of the present study was to characterize the Minnesota dementia and diabetes care landscape by rurality and geographic region. Specifically, we compared the Metro region to five other rural-urban regions. Disease-specific 2017 hospital admission and emergency department (ED) visit data was obtained from the State Center for Health Statistics and the Healthcare Cost and Utilization Project. We used the logistic regression analysis adjusted by multiple covariates to evaluate rural-urban differences in hospital admissions and ED visits. Age-adjusted rates of ED visits for both DM and dementia were significantly higher in rural zip code areas, especially in northeast regions. Rural areas had elevated odds for dementia hospital admissions (OR=1.05, p<0.0001) and ED visits (OR=1.24, p<0.0001), but decreased odds for DM hospital admission (OR=0.96, p<0.0001) and ED visits (OR=0.96, p<0.0001). This was particularly true in the northeast region (relative to Metro regions) where ED visits were less likely due to DM (OR=0.89, p<0.0001) but more likely related to dementia (ORs=1.42, p<0.0001). Geographic differences for ED visits due to DM were greater than those for dementia, with higher rates for rural as compared to urban regions (northeast MN compared to a large metropolitan region). This geographical mismatch between mortality rates and ED visit rates may illustrate the relative lack of access to health services in rural MN.

